# Acute responses to a potentiation warm-up protocol on sprint and change of direction in female football players: a randomized controlled study

**DOI:** 10.1186/s13102-024-01015-z

**Published:** 2024-11-12

**Authors:** Tomás Zylberberg, Ricardo Martins, Svein Arne Pettersen, José Afonso, Ivan André Matias Vale Baptista

**Affiliations:** 1 Center of Research, Education, Innovation, and Intervention in Sport (CIFI2D), Faculty of Sport, University of Porto, Porto, Portugal; 2 Department of Arts, Humanities and Sports, School of Education, Polytechnic Institute of Beja, Beja, Portugal; 3 Department of Physical Education, Faculty of Education Sciences, University of Cadiz, Puerto Real, Cadiz Spain; 4 School of Sport Sciences, Faculty of Health Sciences, UiT The Arctic University of Norway, Tromsø, Norway; 5 Department of Computer Science, Faculty of Science and Technology, UiT The Arctic University of Norway, Tromsø, Norway

**Keywords:** Performance enhancement, Warm-up, Sprint, Change of direction, Women’s football

## Abstract

**Objectives:**

To evaluate the acute effect of exposure to a potentiation warm-up protocol compared to a usual warm-up program.

**Design:**

Randomized parallel control trial.

**Setting:**

Synthetic grass in the club’s facilities (Portugal).

**Participants:**

Seventeen female football players (age: 23.9 ± 3.9 years), were randomly allocated to a control (*n* = 8) and an experimental group (*n* = 9). To allocate the players, a table was computer-generated by a research team member with no involvement in the trial.

**Intervention:**

The control group performed their usual warm-up program, while the experimental group performed a potentiation warm-up protocol with jumps combined with sprints with change of direction.

**Main outcomes measures:**

The players were tested pre- and post-intervention for a 40-m linear sprint and pre-planned change of direction using the T-test.

**Statistical analysis:**

An intention-to-treat analysis was performed, with all the participants originally randomized being involved. The normal distribution was verified by the Shapiro-Wilk test. The assumption of sphericity was analyzed. Effect sizes were calculated using partial eta squared.

**Results:**

No significant pre-post differences in the T-test and in the 40-m sprint were detected for any group. However, in the T-test, large effect sizes in time increments were observed within the experimental (0.27 s; *p* > 0.05, η_p_^2^ = 0.176) and control groups (0.06 s; *p* > 0.05, η_p_^2^ = 0.176). Also, in the 40-m sprint, large effect sizes in time increments were observed within the experimental (0.05 s; *p* > 0.05, η_p_^2^ = 0.251) and control groups (0.09 s; *p* > 0.05, η_p_^2^ = 0.251).

**Conclusions:**

The performance-enhancing ability of the potentiation method performed at warm-up was not verified when applied to female football players. Thus, the potentiation methods may not improve sprint and COD ability for this population. However, the lack of statistical significance may have been due to reduced statistical power, as three of four effects suggest acute performance impairment after a supposed potentiation-oriented warm-up. Nevertheless, the presence of a statistical type 2 error cannot be ruled out.

**Registration number (retrospectively registered):**

NCT06555185; Project URL: https://clinicaltrials.gov/study/NCT06555185.

**Supplementary Information:**

The online version contains supplementary material available at 10.1186/s13102-024-01015-z.

## Background

A warm-up has the potential to enhance physical qualities for better athletic performance in training and competition [1]. Warming up before training sessions is expected to increase body temperature [2] and physical readiness [1, 3], and provides an opportunity to mentally prepare for the training contents through strengthening concentration and self-confidence [4]. Previous research suggested that warm-up protocols should have dynamic movements that increase body temperature and range of motion, enhance motor unit excitability, improve kinesthetic awareness and work on technique by reinforcing critical motor programs [5]. Recently, warm-up protocols aiming to improve acute performance and employing potentiation methods have been proposed in the literature [1, 6]. These potentiation methods are characterized by a variety of exercises exploring the stretching-shortening cycle [7], promoting the ability of the neural and musculotendinous systems to produce maximum power [8].

Regarding the potentiation effects, Post-Activation Potentiation (PAP) and Post-Activation Performance Enhancement (PAPE) are two of the most studied phenomena, however, some differences distinguish these two mechanisms [9]. The phenomenon of PAP is associated with an increase in contractile muscle force in response to maximal voluntary contractions, enhancing subsequent muscle contractions, measured as the maximal contraction force evoked by supramaximal electrical stimulation [9]. The literature suggests a time window to optimize the PAP, with a recovery period between 1- to 3-minutes (min) [10]. Conversely, PAPE refers to the increased maximal voluntary strength, power, or velocity (i.e., dynamic or isometric) following a conditioning contraction [9]. Accordingly, different training protocols have been used, such as plyometric exercises [6], resistance training [11], sprint training [12], the flywheel paradigm [13], and change of direction (COD) drills [14]. For PAPE, the performance-enhancing effects may be observed after a resting period, peaking 3- to 10-min after a conditioning contraction [10, 15].

Bearing in mind that traditional warm-ups may also enhance subsequent performance [3], even if not specifically targeting PAP or PAPE, we will adopt a broader concept of potentiation warm-up, i.e., a general acute benefit for subsequent performance. Additionally, despite being well-studied in men [7, 14], the data available on acute potentiation effects of warm-up should be carefully interpreted for women, due to major anthropometrics, strength, and fitness differences between both sexes [16, 17], large disparities in explosive actions [18], apart from the greater performance variability observed in female compared to male athletes [19].

However, the effectiveness of these protocols depends on the balance between fatigue and potentiation [20, 21], which is affected by a variety of factors, including training experience [9], resting time using an optimal time-window [20], the intensity of the conditioning activity performed [22], the volume of warm-up [1], muscle fibre type and sex [23]. Therefore, this study aims to determine if the performance of female football players is affected after the exposure to a potentiation protocol. It is hypothesized that performance in the selected physical tests will improve significantly and meaningfully after performing the chosen potentiation protocol compared to the control group’s warm-up.

## Methods

### Trial design

This study followed the CONSORT guidelines, using a randomized parallel trial in a single highly trained female football team classified as Tier 3 (i.e., highly trained/national level) [24], competing in the Portuguese top division. Players were recruited at the beginning (September) of the 2022/23 season and the follow-up was carried on the second half (March-April) of the 2022/23 season. A convenience and purposive sampling were used, and the participants were randomized into an experimental (EXPG, potentiation warm-up) and a control group (CONG, team’s usual warm-up). The EXPG had a familiarization week with the potentiation protocol prior to the intervention period, while the CONG continued the usual warm-up program. Both groups were tested before and after performing the warm-up. The physical tests and intervention protocol took place during the competitive period.

All training sessions and physical tests were performed on the synthetic grass in the club’s facilities (temperature, 15–20 °C; wind, 5,6–22,2 km/h). The tests were applied by members of the technical staff, who were not blinded to the interventions. The technology used was objective and reliable, hardly influenced by knowledge of the interventions to which the participants were assigned [25].

This research was approved by the ethical committee of the Faculty of Sport of the University of Porto, with the following approval code assigned CEFADE 03 2022 and performed in accordance with the ethical standards of the Declaration of Helsinki (64th WMA Assembly, Fortaleza, Brazil, 2013).

### Participants

**Fig. 1:**
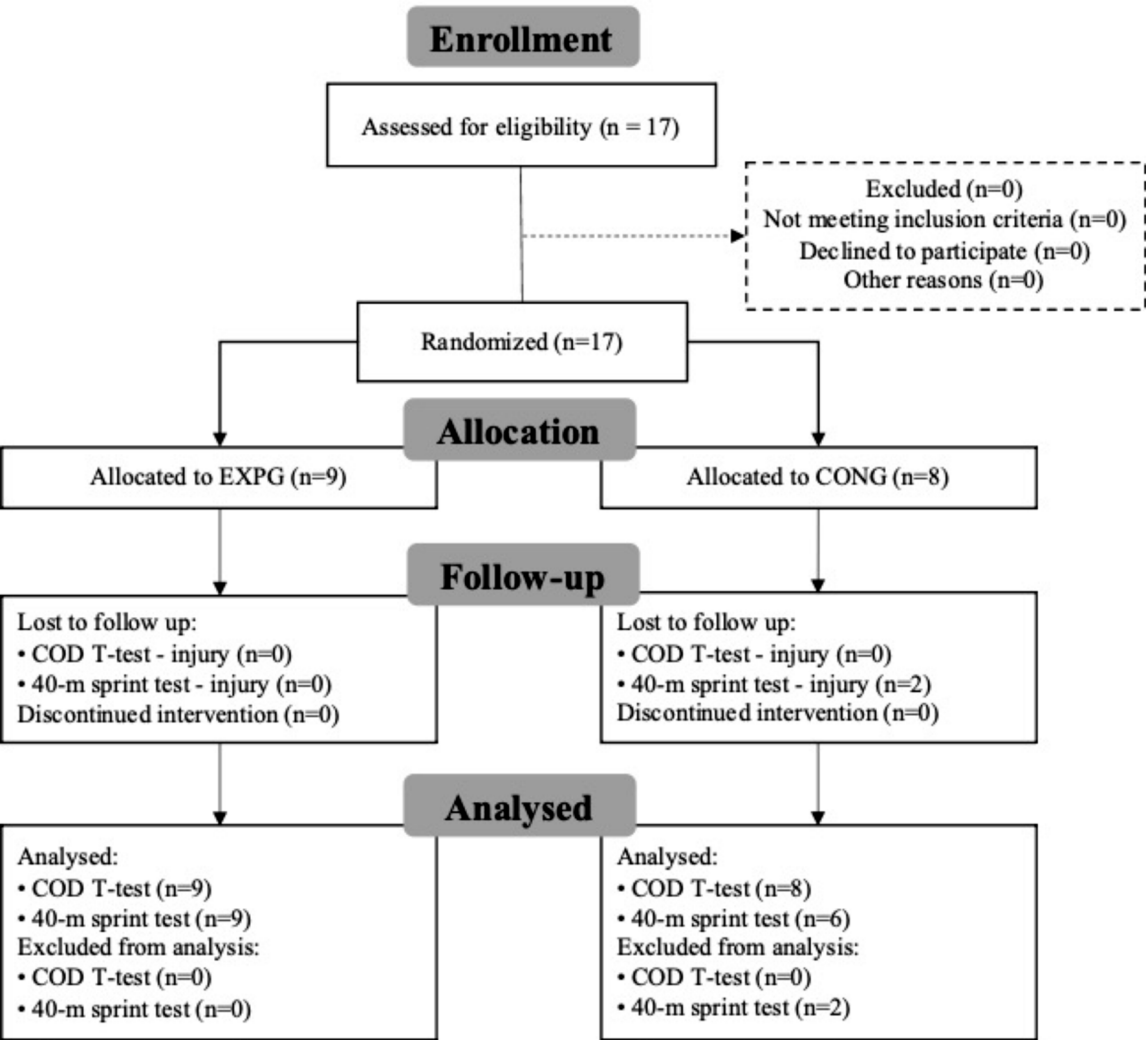
Participants flow diagram. CONG - Control group; EXPG - Experimental intervention group

Seventeen female football players (23.9 ± 3.9 years of age, range 18 to 31; 12.2 ± 4.7 years of experience, range 6 to 23) were randomly allocated between a CONG (*n* = 8) and an EXPG (*n* = 9), using the Excel Software and the Random function, by a member of the research team with no involvement in the trial. The allocation sequence was concealed from the researcher implementing protocols until the beginning of the interventions. Inclusion criteria: players from any playing position (except goalkeepers, by their coaches’ decision) able to train without limitations. Subjects were excluded if they had any existing medical conditions that could compromise participation. All participants were informed about the purpose, content, and potential risks and benefits of the study, and signed an informed consent. In the CONG, two of the eight eligible athletes did not perform the 40-m sprint test due to muscle discomfort during that test. In EXPG, all the players completed all the assessments (Fig. 1). Sample power calculations were not performed for this study, due to the reduced number of players available in the female football team, and all players available were recruited for the study.

### Experimental and active controls

**Fig. 2:**
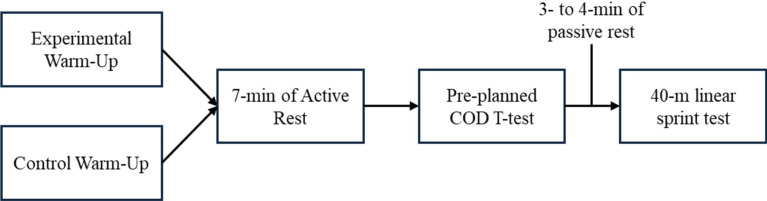
Experimental design of the study

The interventions lasted three weeks. During the first week, all players involved in the study were familiarized with the physical tests, which were performed before training sessions (T-test always before the 40-m sprint test). The intervention was carried out in the following two weeks, in a total of four training sessions (i.e., twice a week). The data collection of the physical tests started in the second week. The initial data collection took place at the onset of the first training session. The second data collection was executed at the conclusion of the third week, following the conclusion of the fourth protocol session. During the testing sessions both groups rested actively for 7-min before starting the T-test, and more 3- to 4-min of passive rest until starting the 40-m linear sprint, as presented in Fig. 2. The duration of each session was ~ 20-min for both groups.

The intervention used a modified version of a previously used protocol [14], while the CONG followed their usual warm-up. Both groups engaged in warm-up sessions of identical duration, conducted at the same time of day, with the participants wearing their normal football shoes. Both warm-up protocol designs are detailed in Table 1.

**Table 1:** Experimental and control warm-up design

Experimental warm-up design				
Sets	1st Set	2nd Set	3rd Set	4th Set
Exercises	6 hurdle jumps + 15-m sprint with COD	6 lateral hurdle jumps + 10-m sprint with COD	6 bouncy strides + 15-m sprint with COD	6 broad jumps + 10-m sprint with COD
Repetitions and rest interval between repetitions	3 repetitions (90s rest between repetition)	3 repetitions (90s rest between repetition)	3 repetitions (90s rest between repetition)	3 repetitions (90s rest between repetition)
Rest interval between sets	90s of rest between sets			
**Control warm-up design**				
Sets	1st phase		2nd phase	
Exercises	Slow jogging + light skipping + dynamic stretches		Football-specific exercises with and without ball, with accelerations, COD, jumping hurdles and ball possession games	
Duration	5 min		15 min	
Rest between phases	Light active rest (jogging) during the transition time between phases			

Note: COD – Change of direction

### Outcomes

#### T-test

Pre-planned COD was assessed through the T-test [26], and applied according to standardized procedures described elsewhere [27]. However, forcing the participant to touch on each cone is not uniform [28], and this step was withdrawn. We implemented course directions used previously [27]. The validated and reliable WICHROⓇ Wireless photocell system (ChronojumpⓇ), which consists of two pairs of barriers containing the photocell and its double reflector, was used [29], and the test started and ended on the same pair of barriers. Each player performed each test once due to the time limitation for collecting the test data.

#### 40-m linear sprint

The same WICHROⓇ Wireless photocell system was used for the 40-m linear sprint [30]. The time was counted in seconds (s) and thousandths of a second (ms) with an error of ± 0.001 s [31]. After a 5 s countdown, the participants ran forward following the route marked by cones, one at 0 m indicating the start place and the other at 40 m indicating the finish place. Participants started the test from a standing start position with the front foot approximately 2 cm behind the first cone.

### Statistical analysis

An intention-to-treat analysis was performed, with all the participants originally randomized being involved. The normal distribution was verified by the Shapiro-Wilk test. The statistical analysis involved descriptive statistics, inferential statistics, and data are presented by mean ± standard deviation. and then a mixed repeated measures ANOVA (i.e., within- and between-factors) was used. The assumption of sphericity was analyzed. Effect sizes were calculated using partial eta squared (η_p_^2^) and will be classified as small (0.010–0.059), moderate (0.060–0.137), and large (> 0.137) [32]. Statistical significance was assumed at *p* < 0.05. Statistical analysis was performed using the IBM SPSS (*Statistical Package for the Social Sciences*) version 27.

## Results

Results obtained in the pre-planned COD T-test and in the 40-m sprint test, for both groups, are presented in Table 2. The ANOVA analysis indicated neither a significant time nor group vs. time interaction effect, nor between groups for both tests.

### T-test

In the pre-planned COD T-test, the decrements in performance observed in the EXPG (0.27 s) and the CONG (0.06 s) were not statistically significant, however, it presented a main time effect with a large effect size (*p* > 0.05, η_p_^2^ = 0.176).

### 40-m Sprint Test

In the 40-m sprint test, it was observed non-significant decrements in performance in the EXPG (0.05 s) and in the CONG (0.09 s). However, it presented a main time effect with a large effect size (*p* > 0.05, η_p_^2^ = 0.251).

**Table 2:** Comparison between pre- and post-protocol performance in the 40-m sprint test and the pre-planned COD t-test for EXPG and CONG

Test	Groups	Pre-Protocol (s)	Post- Protocol (s)	Change (s)	∆ (%)	p-value			ES
						Time	G×T	Group	Time η_p_^2^
Pre-planned COD t-test	EXPG	8.95 ± 0.27	9.22 ± 0.24	0.27 ± 0.26	2.93	0.094	0.258	0.555	0.176
	CONG	8.97 ± 0.52	9.03 ± 0.31	0.06 ± 0.48	0.66				
40-m sprint test	EXPG	6.17 ± 0.24	6.22 ± 0.25	0.05 ± 0.08	0.80	0.057	0.548	0.253	0.251
	CONG	6.02 ± 0.12	6.11 ± 0.17	0.09 ± 0.18	1.47				

Note: Statistical analysis of mixed repeated measures ANOVA 2(group) × 2(time). Values presented as mean ± SD; p - significance value of the mixed repeated measures ANOVA test; η_p_^2^ – partial eta squared; CONG - Control group; EXPG - Experimental intervention group; G×T – interaction between group and time; ES – effect size

## Discussion

To our knowledge, this was the first study to examine the effects of a potentiation protocol performed in the warm-up on the sprint and COD ability in highly trained female football players. The main results do not support our initial hypothesis, showing that the potentiation warm-up protocol failed to enhance the performance in the speed and COD tests, and indeed likely generated more fatigue than potentiation. Despite lack of statistical significance, effect sizes supported performance decrements post-warm-up.

A systematic review which examined the effects of PAPE on sprint and COD performance in athletes from different sports observed that only one study revealed strong evidence that such protocols may improve sprint performance and limited evidence for improvements in COD ability [33]. However, this study consisted of collegiate women that were merely physically active. The authors also reported that such enhancements have large individual variations in responses and are context-dependent, requiring further research to draw clear conclusions, especially in COD ability [33]. Therefore, an imbalance between fatigue and potentiation should be considered when interpreting the presented results, as well as the familiarization time with the protocol and the total volume of pre-conditioning exercise performed that might play a key role.

A direct comparison of our results to similar studies is hampered because research is often conducted in males. In the study of Aloui et al. [14], 34 male football players showed improvements in both sprint performance and COD ability after being exposed to a potentiation warm-up protocol for eight weeks. However, these results may not be comparable to our study due to possible chronic adaptations, which may explain the improvements in contrast to the results of our study. Another study using potentiation methods through trunk jumps, linear sprints and COD drills performed as a re-warm-up strategy, acutely enhanced the COD ability assessed by the T-test, however, this intervention was also carried out in male soccer players [34]. The study of Zisi et al. [35] involving sprinters from both sexes, investigated the effects of plyometric training on 30-m sprint test, and the results revealed an improvement in the subsequent performance of the acceleration phase of the 5-m and 10-m sprint by 1.7% and 1.1%, respectively, compared to baseline. However, non-significant results were observed in the 30-m sprint test between groups, which is parallel to our results, since a protocol that included plyometric training was also followed.

Some of the contrasting results may be explained by potential differences in response to the training stimuli between sexes, where men tend to exert higher potentiation effects [36, 37], and the transitory enhanced performance promoted by potentiation strategies also tends to be shorter in women, limiting its use in sports competitions [38]. A possible mechanism for the lower potentiation effect on women may be due to a lower type-II muscle fibre composition when compared to men [37]. Moreover, the study used as reference for designing the experimental protocol was developed for young male football players [14], and such a training program may not be the most appropriate for female athletes. A recent systematic review on COD improvements through plyometric training [39] suggested an ideal protocol of 2 weekly sessions for 6–9 weeks with a rest interval between sessions of 48–72 h, and a training volume of 1–6 exercises at maximum intensity with a rest period between sets of 60–90 s, which is similar (except the number of weeks) to the protocol adopted in our study. Even though the experimental protocol used in the present study involved a multicomponent training program, where plyometric exercises were combined with acceleration and COD work, this combination of different training components has previously been tested in male athletes [7, 40], while maintaining the same plyometric training volume recommended for plyometric-exclusive training programs. However, such training volumes and rest times are likely unsuitable for female since they were specifically designed for male athletes, and further studies with female populations are requested for the development of more sex-specific potentiation protocols.

As the results indicated a large effect size in diminishing subsequent performance after the potentiation warm-up, the lack of significant differences may be attributed to the reduced sample size. Moreover, the sample being underpowered may have result in false negatives (i.e., non-detection of significant differences due to poor power). The hypothesis of acute fatigue should also be raised as a potential cause, as it may explain the impaired neuromuscular performance in such protocols [21, 41, 42]. However, it is difficult to draw strong conclusions regarding this cause due to the several factors that impact potentiation effects [20, 21, 33, 41, 42].

On the other hand, considering the results obtained in this study, the most plausible reasons for the results may be related to the small sample size, the time frame of exposure, and the total volume of exercises, with the last two being suggested as determinant factors in the success of interventional protocols [43]. Nevertheless, applied research with national-level (or higher) players is scarce, particularly in women, and therefore, studies with this type of participants are of utmost importance, even if involving small sample sizes. Correspondingly, regarding the time frame between the potentiation activity and the tests performed in this study, it might have been too long to induce performance improvements, considering that more recent literature suggests a 2- to 8-min recovery interval [15]. This limitation has a significant impact, especially since the participants are women, who experience an earlier loss of the potentiation effect compared to men [38].

In addition, our work does not allow us to conclude whether the potentiation protocol elicited a level of acute fatigue that explains the decrement in performance, due to a lack of an additional experimental group performing the same potentiation protocol but under a lower dose. Therefore, future research should evaluate this scenario and more studies with a larger sample size and various experimental groups are needed to corroborate the findings of the present study regarding the acute effects of potentiation methods in female football players.

## Conclusion

The performance-enhancing ability of the potentiation method performed at warm-up was not verified when applied to female football players. Thus, for this population, the potentiation methods may not be effective in improving sprint and COD ability. However, the competitive phase, volume of training, fatigue induced by the potentiation method and recovery time should be considered when coaches plan to apply such types of protocols aiming to improve performance acutely. The selection of a proper ratio of training volume and rest time is a crucial factor for the usefulness of potentiation warm-ups in female athletes. Therefore, for the development of further potentiation warm-up protocols, it seems plausible to consider lower volumes and longer rest periods than previously used in male athletes, to avoid eventual deleterious effects generated by excessive fatigue.

## Electronic supplementary material

Below is the link to the electronic supplementary material.

Supplementary Material 1

## Data Availability

The datasets generated and analysed during the current study are available in the Mendeley Data repository, https://data.mendeley.com/datasets/2cwcz2vx4v/1.

## Abbreviations

COD: Change of direction
CONG: Control group
EXPG: Experimental group
PAP: Post-activation potentiation
PAPE: Post-activation performance enhancement

## References

[1] Afonso J, Brito J, Abade E, et al. Revisiting the ‘Whys’ and ‘Hows’ of the Warm-Up: are we asking the right questions? Sports Med. 2024;54(1):23–30. 37658965 10.1007/s40279-023-01908-y PMC10798919

[2] Kapnia A, Dallas CN, Gerodimos V, et al. Impact of Warm-Up on muscle temperature and athletic performance. Res Q Exerc Sport. 2023;94(2):460–5. 35412960 10.1080/02701367.2021.2007212

[3] Thapa RK, Clemente FM, Moran J, et al. Warm-up optimization in amateur male soccer players: a comparison of small-sided games and traditional warm-up routines on physical fitness qualities. Biol Sport. 2023;40(1):321–9. 36636187 10.5114/biolsport.2023.114286 PMC9806743

[4] Fujii N, Fujisawa K, Dobashi K, et al. Effects of High-Intensity Exercise Repetition Number during warm-up on physiological responses, perceptions, readiness, and performance. Res Q Exerc Sport. 2023;94(1):163–72. 34699333 10.1080/02701367.2021.1950901

[5] Thompsen AG, Kackley T, Palumbo MA, et al. Acute effects of different warm-up protocols with and without a weighted vest on jumping performance in athletic women. J Strength Cond Res. 2007;21(1):52–6. 17313270 10.1519/00124278-200702000-00010

[6] Brink NJ, Constantinou D, Torres G. Postactivation performance enhancement (PAPE) using a vertical jump to improve vertical jump performance. J Sports Med Phys Fit. 2022;62(11):1419–26. 10.23736/S0022-4707.22.12899-9 35084163

[7] Michailidis Y, Tabouris A, Metaxas T. Effects of Plyometric and directional training on physical fitness parameters in Youth Soccer players. Int J Sports Physiol Perform. 2019;14(3):392–8. 30204520 10.1123/ijspp.2018-0545

[8] Wang YC, Zhang N. Effects of plyometric training on soccer players. Exp Ther Med. 2016;12(2):550–4. 27446242 10.3892/etm.2016.3419 PMC4950532

[9] Prieske O, Behrens M, Chaabene H, et al. Time to Differentiate Postactivation Potentiation from Performance Enhancement in the strength and Conditioning Community. Sports Med. 2020;50(9):1559–65. 32495254 10.1007/s40279-020-01300-0 PMC7441077

[10] Blazevich AJ, Babault N. Post-activation potentiation Versus Post-activation Performance Enhancement in humans: historical perspective, underlying mechanisms, and current issues. Front Physiol. 2019;10. 10.3389/fphys.2019.01359 PMC6838751 31736781

[11] Carbone L, Garzón M, Chulvi-Medrano I, et al. Effects of heavy barbell hip thrust vs back squat on subsequent sprint performance in rugby players. Biol Sport. 2020;37(4):325–31. 33343065 10.5114/biolsport.2020.96316 PMC7725042

[12] Bevan HR, Cunningham DJ, Tooley EP, et al. Influence of Postactivation potentiation on sprinting performance in Professional Rugby players. J Strength Conditioning Res. 2010;24(3):701–5. 10.1519/JSC.0b013e3181c7b68a 20145565

[13] Sañudo B, de Hoyo M, Haff GG et al. Influence of Strength Level on the Acute Post-activation Performance Enhancement following Flywheel and Free Weight Resistance Training. Sens (Basel). 2020;20(24). 10.3390/s20247156 PMC7764837 33327405

[14] Aloui G, Hermassi S, Hayes LD, et al. Loaded plyometrics and short sprints with change-of-direction training enhance jumping, sprinting, agility, and Balance Performance of Male Soccer players. Appl Sci. 2021;11(12):5587.

[15] Gautam A, Singh P, Varghese V. Effects of Postactivation Potentiation enhacement on sprint and change-of-direction performance in athletes: a systematic review. J Bodyw Mov Ther. 2024;39:243–50. 38876634 10.1016/j.jbmt.2024.02.006

[16] Emmonds S, Dalton Barron N, Myhill N, et al. Locomotor and technical characteristics of female soccer players training: exploration of differences between competition standards. Sci Med Footb. 2023;7(3):189–97. 35703123 10.1080/24733938.2022.2089723

[17] Mujika I, Santisteban J, Impellizzeri FM, et al. Fitness determinants of success in men’s and women’s football. J Sports Sci. 2009;27(2):107–14. 19058090 10.1080/02640410802428071

[18] de Araújo MC, Baumgart C, Jansen CT, et al. Sex differences in physical capacities of German Bundesliga soccer players. J Strength Conditioning Res. 2020;34(8):2329–37. 10.1519/JSC.0000000000002662 29927885

[19] Baptista I, Winther AK, Johansen D, et al. The variability of physical match demands in elite women’s football. Sci Med Footb. 2022;6(5):559–65. 35060844 10.1080/24733938.2022.2027999

[20] Tillin NA, Bishop D. Factors modulating post-activation potentiation and its effect on performance of subsequent explosive activities. Sports Med. 2009;39(2):147–66. 19203135 10.2165/00007256-200939020-00004

[21] Wilson JM, Duncan NM, Marin PJ, et al. Meta-analysis of postactivation potentiation and power: effects of conditioning activity, volume, gender, rest periods, and training status. J Strength Cond Res. 2013;27(3):854–9. 22580978 10.1519/JSC.0b013e31825c2bdb

[22] Sale DG. Postactivation potentiation: role in human performance. Exerc Sport Sci Rev. 2002;30(3):138–43. 12150573 10.1097/00003677-200207000-00008

[23] DeRenne C. Effects of Postactivation Potentiation warm-up in Male and Female Sport performances: a brief review. Strength Conditioning J. 2010;32(6):58–64.

[24] McKay AKA, Stellingwerff T, Smith ES, et al. Defining training and performance caliber: a participant classification Framework. Int J Sports Physiol Perform. 2022;17(2):317–31. 34965513 10.1123/ijspp.2021-0451

[25] Bastida Castillo A, Gómez Carmona CD, Pino Ortega J, et al. Validity of an inertial system to measure sprint time and sport task time: a proposal for the integration of photocells in an inertial system. Int J Perform Anal Sport. 2017;17(4):600–8.

[26] Krolo A, Gilic B, Foretic N et al. Agility testing in Youth Football (Soccer)players; evaluating reliability, validity, and correlates of newly developed testing protocols. Int J Environ Res Public Health. 2020;17(1). 10.3390/ijerph17010294 PMC6981745 31906269

[27] Miller MG, Herniman JJ, Ricard MD, et al. The effects of a 6-week plyometric training program on agility. J Sports Sci Med. 2006;5(3):459–65. 24353464 PMC3842147

[28] Raya MA, Gailey RS, Gaunaurd IA, et al. Comparison of three agility tests with male servicemembers: Edgren Side Step Test, T-Test, and Illinois agility test. J Rehabil Res Dev. 2013;50(7):951–60. 24301432 10.1682/JRRD.2012.05.0096

[29] López-Samanes Á, Gómez Parra A, Moreno-Pérez V et al. Does Acute Beetroot Juice Supplementation improve Neuromuscular Performance and Match Activity in Young Basketball players? A Randomized, Placebo-controlled study. Nutrients. 2020;12(1). 10.3390/nu12010188 PMC7019528 31936621

[30] Irurtia A, Torres-Mestre VM, Cebrián-Ponce Á et al. Physical fitness and performance in talented & untalented Young Chinese Soccer players. Healthc (Basel). 2022;10(1). 10.3390/healthcare10010098 PMC8775658 35052262

[31] Sánchez-Pay A, Martínez-Gallego R, Crespo M et al. Key physical factors in the serve velocity of male Professional Wheelchair Tennis players. Int J Environ Res Public Health. 2021;18(4). 10.3390/ijerph18041944 PMC7922282 33671337

[32] Cohen J. Statistical Power Analysis for the behavioral sciences. 2nd ed. Hillsdale, NJ: Lawrence Earlbaum Associate; 1988.

[33] Borba D, Ferreira Júnior J, Santos L, et al. Effect of post-activation potentiation in Athletics: a systematic review. Revista Brasileira De Cineantropometria E Desempenho Humano. 2017;19:128–38.

[34] Matsentides D, Christou M, Zaras N. The effects of different re-warm-up strategies on power, changing of direction and ball shooting velocity in well-trained Soccer players. Sports (Basel). 2023;11(9). 10.3390/sports11090169 PMC10535876 37755846

[35] Zisi M, Stavridis I, Bogdanis G, et al. The Acute effects of Plyometric exercises on Sprint Performance and Kinematics. Physiologia. 2023;3(2):295–304.

[36] Arabatzi F, Patikas D, Zafeiridis A, et al. The Post-activation Potentiation Effect on Squat Jump performance: age and sex effect. Pediatr Exerc Sci. 2014;26(2):187–94. 24225048 10.1123/pes.2013-0052

[37] McKiel A, Woods S, Faragher M et al. Optimization of post-activation potentiation in girls and women. Eur J Appl Physiol. 2024. 10.1007/s00421-024-05475-6 38652270

[38] Koźlenia D, Domaradzki J. The sex effects on changes in jump performance following an isometric back squat conditioning activity in trained adults. Front Physiol. 2023;14:1156636. 37123271 10.3389/fphys.2023.1156636 PMC10133540

[39] Jimenez-Iglesias J, Owen AL, Cruz-Leon C et al. Improving change of direction in male football players through plyometric training: a systematic review. Sport Sci Health. 2024.

[40] Makhlouf I, Chaouachi A, Chaouachi M, et al. Combination of agility and plyometric training provides similar training benefits as combined balance and plyometric training in Young Soccer players. Front Physiol. 2018;9:1611. 30483158 10.3389/fphys.2018.01611 PMC6243212

[41] Rassier DE, Macintosh BR. Coexistence of potentiation and fatigue in skeletal muscle. Braz J Med Biol Res. 2000;33(5):499–508. 10775880 10.1590/s0100-879x2000000500003

[42] Till KA, Cooke C. The effects of postactivation potentiation on sprint and jump performance of male academy soccer players. J Strength Cond Res. 2009;23(7):1960–7. 19855319 10.1519/JSC.0b013e3181b8666e

[43] Thorpe RT, Strudwick AJ, Buchheit M, et al. Monitoring fatigue during the In-Season competitive phase in Elite Soccer players. Int J Sports Physiol Perform. 2015;10(8):958–64. 25710257 10.1123/ijspp.2015-0004

